# Supplementary material for: Left ventricular diastolic dysfunction in non-severe chronic obstructive pulmonary disease – a step forward in cardiovascular comorbidome
Source: PLoS One. 2021 Mar 8;16(3):e0247940. doi: 10.1371/journal.pone.0247940 (PMC7939359; doi:10.1371/journal.pone.0247940)
Supplement: Attachment — Submitted filename: response to reviewers f.docx [file pone.0247940.s003.docx]

**Dear Reviewers,**

**We apologise for the delay of our revision. Due to the Covid-19 pandemia we were very busy in the clinic and we have missed the deadline for revision. As you can see it is only minor revision. So we beg for your benevolence.**

Reviewer #1: This is the revised version of a paper which had been reviewed previously by the Journal. The authors have performed extensive revisions. Overall, I think that the authors have made a big effort and have appropriately addressed most of the reviewers’ comments. There are still a few issues.
1. It is stated in the methods that patients with pulmonary hypertension defined as peak TRV >2.8 m/s were excluded. At the same time cut-off this listed as a criterion for LV diastolic dysfunction.

Dear Reviewer, thank you for your remark. One of the exclusion criteria is pulmonary hypertension, defined as peak TRV >2.8 m/s at rest. So none of our patients had TRV>2,8 m/s at rest. However, during exercise 67/104 patients (64%) had stress induced LVDD.

According to the guidelines it is considered positive if all of the following three conditions are met during exercise: average E/e’ > 14 or septal E/e’ ratio > 15, peak TR velocity > 2.8 m/sec and septal e’ velocity < 7 cm/sec.”Recommendations for the Evaluation of Left Ventricular Diastolic Function by Echocardiography: An Update from the American Society of Echocardiography and the European Association of Cardiovascular Imaging. J Am Soc Echocardiogr 2016;29:277-314’’. So it is stress induced TR>2,8 m/s that is obligatory to define stress induced LVDD.
2. I had asked to report work rate indexed to body weight. This has not been done.

Dear Reviewer, thank you for your remark.We have taken this into consideration this time. After recalculating the results per body weight it became obvious that there is no significant difference in work load achieved by the two groups. This is mentioned in the text and corrected in both text and table.

3. Table 4 is hard to understand. What exactly is the dependent variable? This seems to be a mix of linear and logistic regression. In this type of Table, units must not be reported.

Dear Reviewer, thank you for your remark.We have subdivided the table into table .4. (presenting the correlation analysis) and table .5. presenting the multivariate logistic regression analysis.We have deleted the units as you recommend.

**Table.4. Correlation analysis between stress LV E/e’ ratio and the biomarkers, cardio-pulmonary parameters cut-off values of the echocardiographic idices**

| **Correlation analysis** | **p-value** | **correlation coefficient** |
| --- | --- | --- |
| **Echocardiographic parameters** |  |  |
| LV E/A ratio rest | 0.023 | 0.616 |
| RV parasternal diameter | 0.000 | 0.793 |
| RVWT | 0.000 | 0.219 |
| RAVI | 0.000 | 0.875 |
| RV E/A ratio rest | 0.000 | 0.417 |
| **CPET parameters** |  |  |
| Peak Load | 0.730 | 0.957 |
| Peak VE | 0.287 | 0.613 |
| V'O2 | 0.048 | 0.574 |
| AT, V'O2 | 0.021 | 0.216 |
| RER | 0.943 | 0.452 |
| VE/VCO2 slope | 0.026 | 0.612 |
| HR at rest | 0.737 | 0.247 |
| Peak HR | 0.382 | 0.409 |
| CRI | 0.061 | 0.752 |
| O2 pulse | 0.032 | 0.481 |
| HRR at 1 min | 0.041 | 0.763 |
| BR, % | 0.983 | 0.213 |
| ICdyn | 0.037 | 0.043 |
| **Biomarkers** |  |  |
| PG E2 | 0.041 | 0.038 |

**Table.5. Multivariate regression analysis between stress LV E/e’ ratio and the cut-off values of the echocardiographic idices**

| **Multivariable logistic regression analysis** | **p-value** | **OR** | **95% CI** |
| --- | --- | --- | --- |
| RV parasternal diameter | 0.001 | 19.567 | 3.131-22.290 |
| RAVI | 0.000 | 24.061 | 4.485-29.100 |
| RV E/A ratio | 0.007 | 10.853 | 1.913-21.564 |
